# Genome-wide identification and expression pattern analysis of the Aux/IAA (auxin/indole-3-acetic acid) gene family in alfalfa (*Medicago sativa*) and the potential functions under drought stress

**DOI:** 10.1186/s12864-024-10313-2

**Published:** 2024-04-18

**Authors:** Jinqing Zhang, Shuxia Li, Xueqin Gao, Yaling Liu, BingZhe Fu

**Affiliations:** 1 College of Forestry and Prataculture, Ningxia University, Yinchuan, 750021 China; 2 Ningxia Grassland and Animal Husbandry Engineering Technology Research Center, Xixia District, Yinchuan, Ningxia Hui Autonomous Region, Yinchuan, 750021 China; 3 Key Laboratory for Model Innovation in Forage Production Efficiency, Ministry of Agriculture and Rural Affairs, Yinchuan, 750021 China; 4 Inner Mongolia Pratacultural Technology Innovation Center Co, Ltd, Hohhot, 010000 China

**Keywords:** Genome-wide analysis, *Aux/IAA* gene family, Drought stress, Expression analysis, *Medicago sativa*

## Abstract

**Background:**

Auxin/induced-3-acetic acid (Aux/IAA) is an important plant hormone that affects plant growth and resistance to abiotic stresses. Drought stress is a vital factor in reducing plant biomass yield and production quality. Alfalfa (*Medicago sativa* L.) is the most widely planted leguminous forage and one of the most economically valuable crops in the world. *Aux/IAA* is one of the early responsive gene families of auxin, playing a crucial role in response to drought stress. However, the characteristics of the *Aux/IAA* gene family in alfalfa and its potential function in response to drought stress are still unknown.

**Result:**

A total of 41 *Aux/IAA* gene members were identified in alfalfa genome. The physicochemical, peptide structure, secondary and tertiary structure analysis of proteins encoded by these genes revealed functional diversity of the *MsIAA* gene. A phylogenetic analysis classified the *MsIAA* genes into I-X classes in two subgroups. And according to the gene domain structure, these genes were classified into typical *MsIAA* and atypical *MsIAA*. Gene structure analysis showed that the *MsIAA* genes contained 1–4 related motifs, and except for the third chromosome without *MsIAAs*, they were all located on 7 chromosomes. The gene duplication analysis revealed that segmental duplication and tandem duplication greatly affected the amplification of the *MsIAA* genes. Analysis of the Ka/Ks ratio of duplicated *MsAux/IAA* genes suggested purification selection pressure was high and functional differences were limited. In addition, identification and classification of promoter cis-elements elucidated that *MsIAA* genes contained numerous elements associated to phytohormone response and abiotic stress response. The prediction protein–protein interaction network showed that there was a complex interaction between the *MsAux/IAA* genes. Gene expression profiles were tissue-specific, and *MsAux/IAA* had a broad response to both common abiotic stress (ABA, salt, drought and cold) and heavy metal stress (Al and Pb). Furthermore, the expression patterns analysis of 41 *Aux/IAA* genes by the quantitative reverse transcription polymerase chain reaction (qRT-PCR) showed that *Aux/IAA* genes can act as positive or negative factors to regulate the drought resistance in alfalfa.

**Conclusion:**

This study provides useful information for the alfalfa auxin signaling gene families and candidate evidence for further investigation on the role of *Aux/IAA* under drought stress. Future studies could further elucidate the functional mechanism of the *MsIAA* genes response to drought stress.

**Supplementary Information:**

The online version contains supplementary material available at 10.1186/s12864-024-10313-2.

## Introduction

Indole-3-acetic acid (IAA) is the main natural form of auxin in higher plants and plays an important role in biosynthesis, metabolism, transport, location, signal transduction, and crosstalk with other phytohormones [1, 2]. Auxin signal is the transmission of signal in an organism, particularly through cells. The auxin signaling pathway involves several stages such as signal recognition, expression of downstream auxin related genes, and physiological responses in plants, suggesting it is not singular. Plants can rapidly sense and respond to changes in auxin levels involving several major classes of auxin-responsive genes, including the *Aux/IAA* and *ARF* families [3]. Aux/IAA proteins mediate the auxin responses through interaction with ARF transcription factors [4]. Previous studies showed that *Aux/IAA* family genes were necessary for the regulation of various plant development processes. Detailed explanations of *Aux/IAA* molecular mechanisms have been obtained in plant model systems, particularly in *Arabidopsis thaliana* and rice (*Oryza sativa*) [5]. Once auxin concentration is elevated and the auxin receptor receives an auxin signal, Aux/IAA proteins will degraded by the 26S proteasome, thereby activating ARF transcriptional activity and initiating the downstream auxin response gene-cascade expression [6–9]. Therefore, precise regulation of plant growth and development processes by *Aux/IAA* is achieved by finely transforming the spatiotemporal dynamics of auxin levels into gene reprogramming signals. *Aux/IAA* encoded proteins that are localized to the nucleus and has a short half-life [10], so Aux/IAA proteins are short-lived transcriptional regulatory factor composed of four highly conserved domains, encoded by the early auxin response gene family [11]. Domain I acts as a transcriptional repressor of auxin-regulated genes [12]; Domain II is a TIR1/AFB with a conserved nitrogen removal sequence that can recognize the sequence GWPPV and regulate protein stability of Aux/IAA, interacting with the F-box protein TIR1 to rapidly degrade Aux/IAA protein [13]; Domain III contains the βαα region, and the conserved domain IV represents the acidic region [14], and domains III and IV are capable of homologous or heterodimerization with other *Aux/IAA* or *ARF* to regulate the expression of downstream auxin-responsive genes [15].

*Aux/IAA* is involved in the regulation of a variety of cellular and developmental processes including embryogenesis, axis formation, lateral root initiation, leaf expansion, vascular elongation, tropism, inflorescence development and fruit ripening, apical dominance, and defense response to stress [16–18]. Obviously, many typical phenotypes controlled by auxin signaling will be regulated by *Aux/IAA* [19, 20]. For example, Audran-Delalande *et. al* [21]*.* found that inhibition of *SlIAA9* expression led to parthenogenesis, while silencing of *SlIAA27* or *SlIAA17* altered in fruit size, shape and quality [22, 23]. In addition, *Aux/IAA* can regulate plant growth and development by affecting other hormones. For instance, auxin signaling is involved in fruit ripening regulation through the interaction with ethylene signaling, while the *Aux/IAA* family genes are implicated in the interaction between the two [24]. There are obvious differences in auxin content and ethylene release during peach (*Prunus persica*) ripening, accompanied by similar changes in the expression of the key rate-limiting enzyme gene *ACS1* and the auxin synthesis gene *YUCCA11*, suggesting that auxin may be a prerequisite factor for ethylene release during peach ripening [25, 26]. *Aux/IAA* regulates anther dehiscence, affects pollination and floral organ development by altering the change of jasmonic acid (JA) content [27]. The ethylene response factor *ERF3* (ethylene response factor) is upregulated in *taiaa21*, and *ttarf25* is downregulated in tetraploid wheat (*Triticum aestivum*). Further analysis found that the *TaARF25* promotes the transcription of *ERF3*, and *TaERF3* mutation caused reduced grain size and grain weight in tetraploid wheat, indicating that *TaIAA21* negatively regulated wheat grain size and grain weight through *ARF25-ERFs* pattern, thus improving wheat yield [28]. And *AtIAA7* inhibited flowering time by regulating gibberellin (GA) related-genes *GA20ox1* and *GA20ox22* [3]. The *AtARF6-AtIAA8* module regulated floral organ development by affecting JA levels [29]. It is well known that the crucial role of auxin in plants goes far beyond these, and it also plays pivotal regulatory parts in resisting biotic and abiotic stresses, as well as the Aux/IAA proteins are a large family of auxin co-receptors and transcriptional repressors that have a central role in auxin signaling [30], so the versatility of the auxin implies the broad functionality of *Aux/IAA*.

A detailed understanding of abiotic stress tolerance in plants is essential to provide food security in the face of increasingly harsh climatic conditions [31]. The auxin core gene *Aux/IAA* plays a huge role in plant biotic stress. Among numerous environmental stresses, losses due to drought stress are estimated to be at 30% globally [32], and the yield loss of crops caused by drought exceeds the total loss caused by other non biological factors [33]. Under drought conditions, auxin content is reduced, and transcriptional expression of genes in auxin biosynthesis as well as some auxin responsive genes, including *Aux/IAA* genes, are also affected by drought treatment [34]. There are many examples that the *Aux/IAA* genes involve in regulating plant response to drought stress. Under drought and salt stress, proline and chlorophyll content were significantly reduced in rice *osiaa20* lines, and the abscisic acid (ABA) response gene *OsRab21* was down-regulated in *osiaa20* and up-regulated in *OsIAA20* over-expression lines, indicating that *OsIAA20* played an important role in plant drought and salt stress response through ABA signal transduction pathway [35]. *IAA5*, *IAA6*, and *IAA19* in the *Aux/IAA* gene family were maintained glucosinolates (GLS) levels when plants were exposed to drought, while *AtIAA5/6/19* deficiency caused decreased GLS levels and decreased drought tolerance, indicating that *Aux/IAA* genes regulate drought tolerance in *A. thaliana* by regulation of GLS levels [31]. Alfalfa (*Medicago sativa* L.) is the most widely planted perennial legume forage, with high biomass and crude protein content, rich in digestible nutrients and mineral elements, and is known as the "king of forage" [36, 37]. It is widely planted in North America, Asia, and other continents, and is also one of the most economically valuable crops in the world. In the United States, it is second only to wheat, maize (*Zea mays*) and soybeans (*Glycine max*) are the fourth largest cultivated crops [38, 39]. Drought, as one of the main environmental factors affecting alfalfa productivity, is an important factor limiting its cultivation and promotion [40]. *Aux/IAA* gene family is also known to play an important regulatory role in protecting plants against drought stress. Therefore, identification of *Aux/IAA* genes in tetraploid cultivated alfalfa at the genome-wide level and doing related analysis, as well as analysis of expression patterns under drought stress can provide more comprehensive data for finding a new and broader layer of auxin signaling regulatory network. Sequence characteristics, genomic organization, cis-regulatory elements, and evolutionary duplication, as well as the transcriptional expression patterns of *Aux/IAA* homologous genes under different tissue/developmental stages and abiotic stress conditions were analyzed in the study. Furthermore, we further analyzed the expression levels of 41 *MsIAA* genes under drought stress. These data help to further elucidate the accurate biological functions of the *Aux/IAA* genes in alfalfa and provide basic data for drought resistance breeding.

## Results

### Identification of *MsIAA* genes family and physicochemical properties analysis of the MsIAA proteins

A total of 41 *MsIAA* sequences were identified in the alfalfa genome and named *MsIAA1*—*MsIAA41* according to the order of their occurrence in the genome (Table S1). Analyze the physicochemical properties of the encoded proteins of 41 *MsIAA* genes, including number of amino acids, molecular weight, theoretical isoelectric point, instability index, aliphatic index and grand average of hydropathicity (Table S2). Significant differences exist in the physicochemical properties of the 41 MsIAA proteins, among which, the numbers of amino acids ranged from 59 (MsIAA11) to 1129 (MsIAA14); The molecular weight ranged from 6802.78 Da (MsIAA11) to 126,377.93 Da (MsIAA14). The theoretical isoelectric point were between 4.33 (MsIAA11) and 9.42 (MsIAA2), and the theoretical isoelectric point of 28 MsIAA proteins were less than 7.5, which belonged to acidic proteins and the remaining 13 belonged to alkaline proteins. The instability index of 41 MsIAA family proteins varies between 16.24 (MsIAA29) and 72.96 (MsIAA34), and in which, the instability index of most proteins (31) are more than 40, indicating that they are unstable proteins and the remaining 10 being stable. Except for MsIAA2 with an aliphatic index of 121.26, the aliphatic index of the remaining 40 MsIAA proteins range from 61.23 (MsIAA25) to 95.42 (MsIAA29), all less than 100 and are hydrophilic proteins. With the help of the protein grand average of hydropathicity, 22 MsIAA proteins, whose grand average of hydropathicity is less than -0.5, are hydrophilic proteins, and the other 19 MsIAA proteins are between -0.5 and 0.5, belonging to amphiphilic proteins.

### Subcellular localization, signal peptides, leading peptides and transmembrane structural analysis of MsIAA proteins

**Fig. 1:**
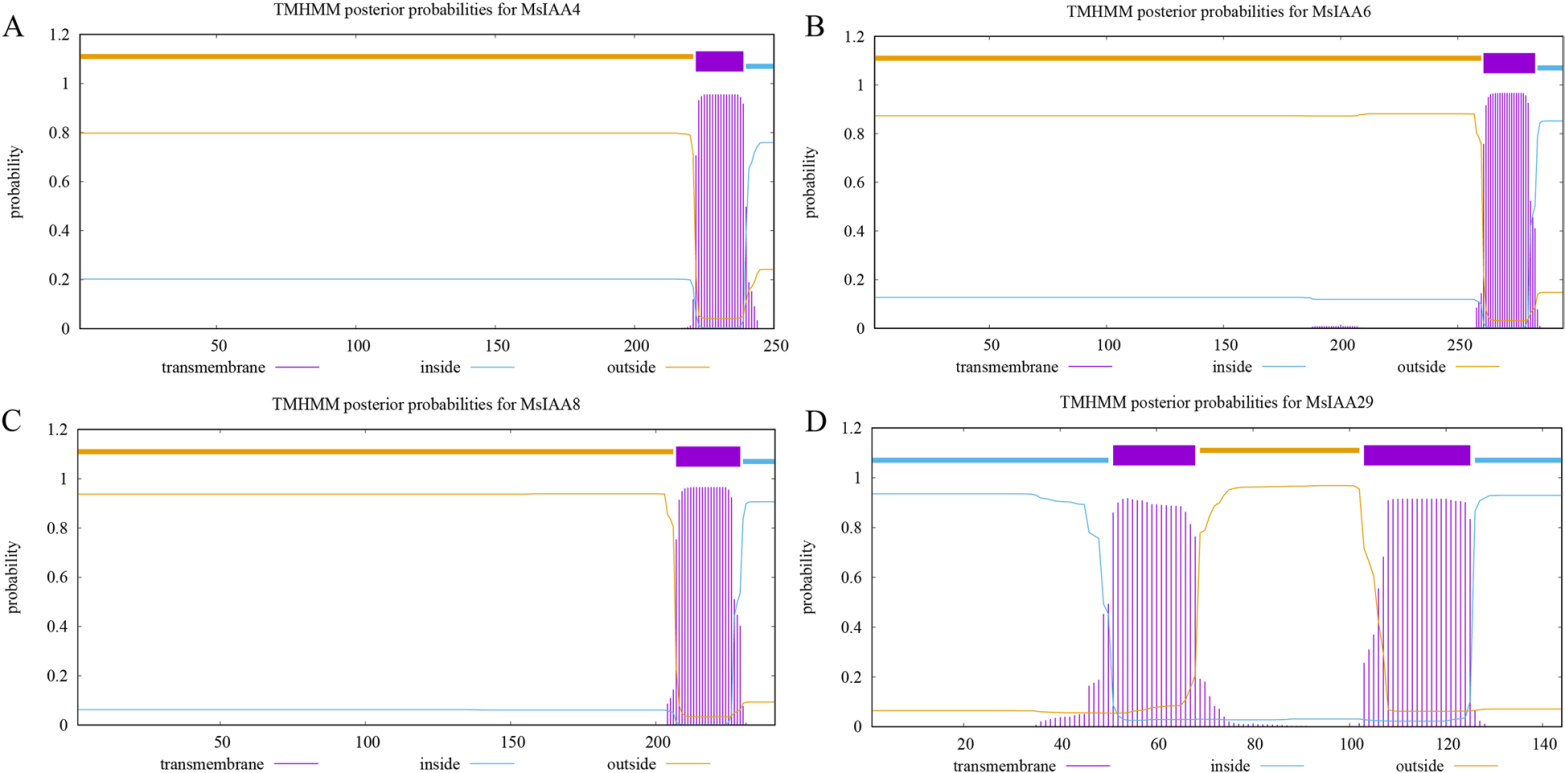
The transmembrane structure analysis of MsAux/IAA proteins. **A**: MsIAA4; **B**: MsIAA6; **C**: MsIAA8; **D**: MsIAA29

The subcellular localization prediction of the 41 MsIAA proteins revealed that most of the MsIAA proteins (MsIAA1, 3, 5, 7, 13–23, 25–28, 30, 31, 33–35, 38) were located in the nucleus, while the other 9 proteins (MsIAA4, 6, 8, 10, 12, 29, 36, 40, 41) were located in the chloroplast, 6 proteins (2, 9, 11, 32, 37, 39) were located in the cytoplasm, and only the MsIAA24 was located in the extracellular matrix (Table S3). Addition to, the results of signal peptide analysis showed that all MsIAA proteins have no peptides and signal peptides. However, analysis of transmembrane structure showed that four proteins (MsIAA4, MsIAA6, MsIAA8, and MsIAA29) had typical transmembrane structures, with MsIAA29 having two typical transmembrane structures and the other three having only one segment each. Interestingly, we found that all four genes are localized in the chloroplast, indicating that these four genes can act across the chloroplast membrane both inside and outside (Fig. 1). On the other hand, the 25 *MsIAA* genes located in the nucleus serve as transcription factors without any signaling peptides, leading peptides, or transmembrane structures, so they do not secrete or transport and only function within the nucleus.

### Analysis of the secondary and tertiary structure of the MsIAA proteins

**Fig. 2:**
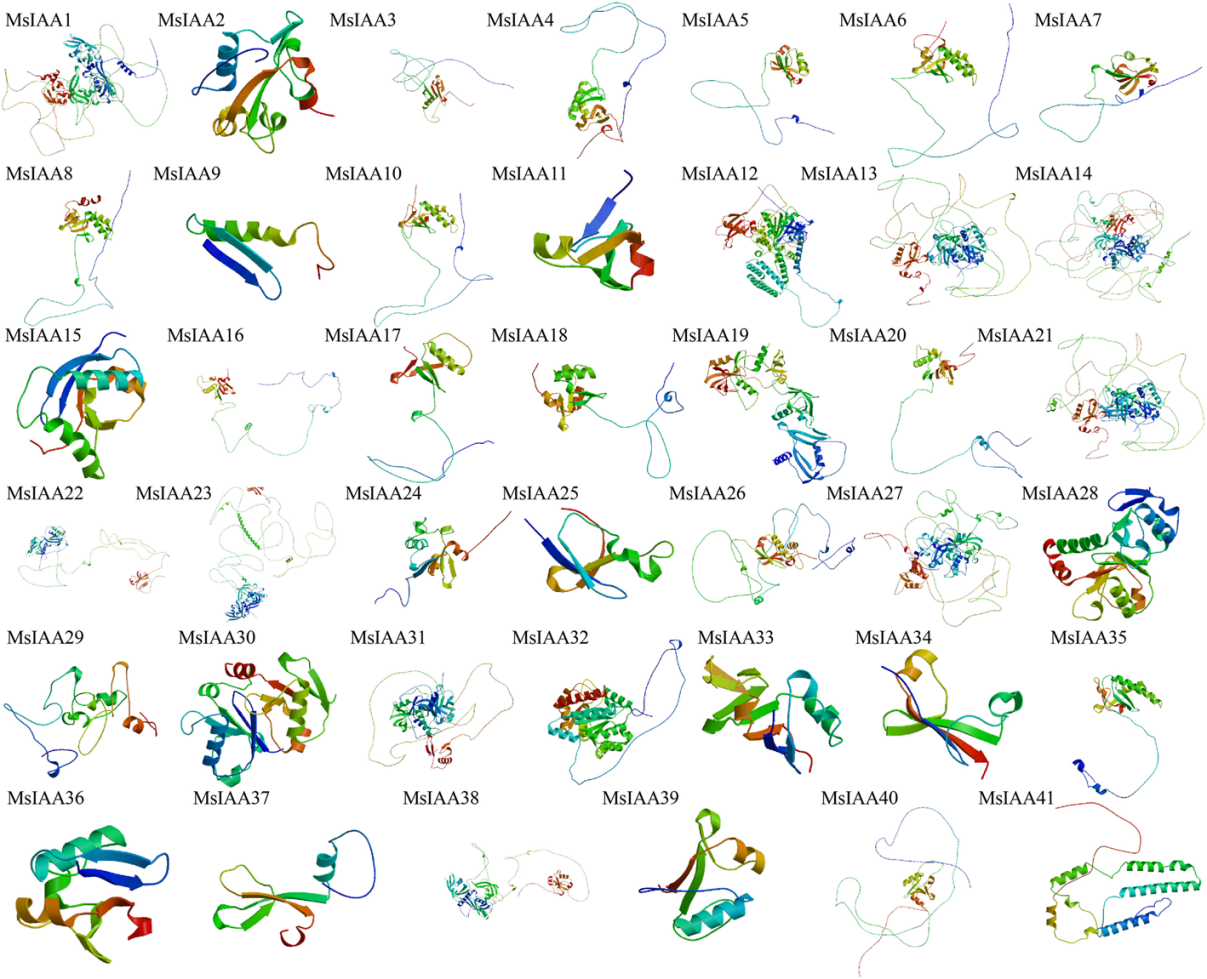
Analysis of tertiary structures of 41 MsAux/IAA proteins

Analysis of the secondary structure of MsIAA protein, the results found that, except for the extended strand of MsIAA11 protein, alpha helix of MsIAA12, 33, the secondary structures of remaining 37 MsIAA proteins, the largest proportion was random coil, as well as the composition percentage of 34 proteins was shown as random coil > alpha helix > extended strand > beta turn (Table S3). At the same time, we also constructed a tertiary structure of MsIAA protein, which showed significant differences due to different percentages of secondary structure composition. Total of 41 MsIAA proteins each have specific spatial structural types, and no two completely consistent spatial configurations have been observed (Fig. 2). Structure determines the function, so we speculate that its unique spatial configuration may be related to its unique function.

### Phylogenetic analysis of IAA proteins from *A. thaliana*, *G. max*, *Medicago truncatula* and *M. sativa*

**Fig. 3:**
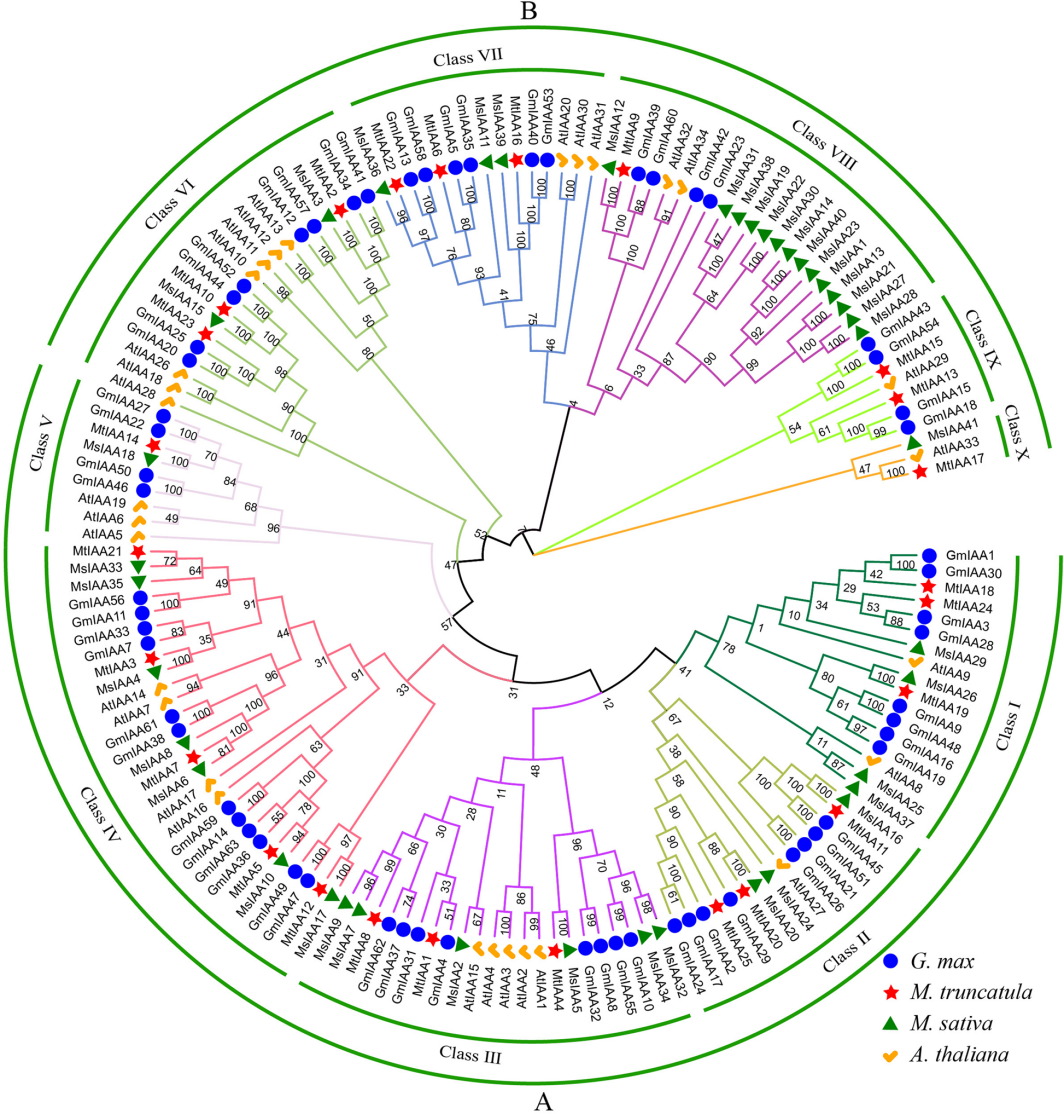
Phylogenetic analysis of Aux/IAA proteins from *Arabidopsis thaliana*, *Glycine max, Medicago truncatula* and *Medicago sativa*

Phylogenetic relationship analysis was conducted on 158 *IAA* family members from 4 species (25 for *M. truncatula*, 63 for soybeans, 29 for *Arabidopsis*, and 41 for alfalfa). The results showed that the IAA family members were clearly divided into two groups: A and B, and further subdivided into 10 classes. Class I-V belongs to group A and consists of 91 members (15 for *Arabidopsis*, 41 for soybeans, 15 for *M. truncatula*, and 21 for alfalfa); Class VI-X belongs to group B and consists of 72 members (14 *Arabidopsis*, 22 soybeans, 10 M*. truncatula*, and 20 M*. sativa*). Except for Class IX, which does not include members of *M. sativa*, the remaining classes all contain IAA members of the four species (Fig. 3).

### Analysis of conserved motif and gene structure of the *MsIAA* family members

**Fig. 4:**
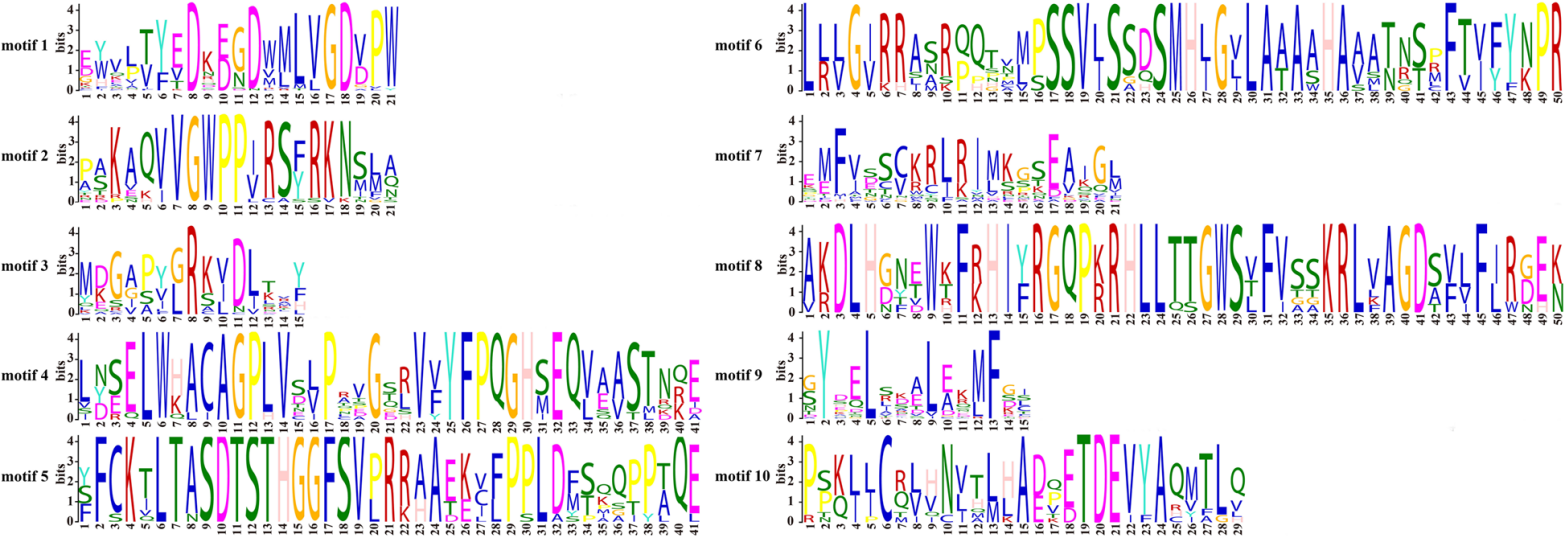
Structural analysis of conserved Motif of MsAux/IAA proteins

**Fig. 5:**
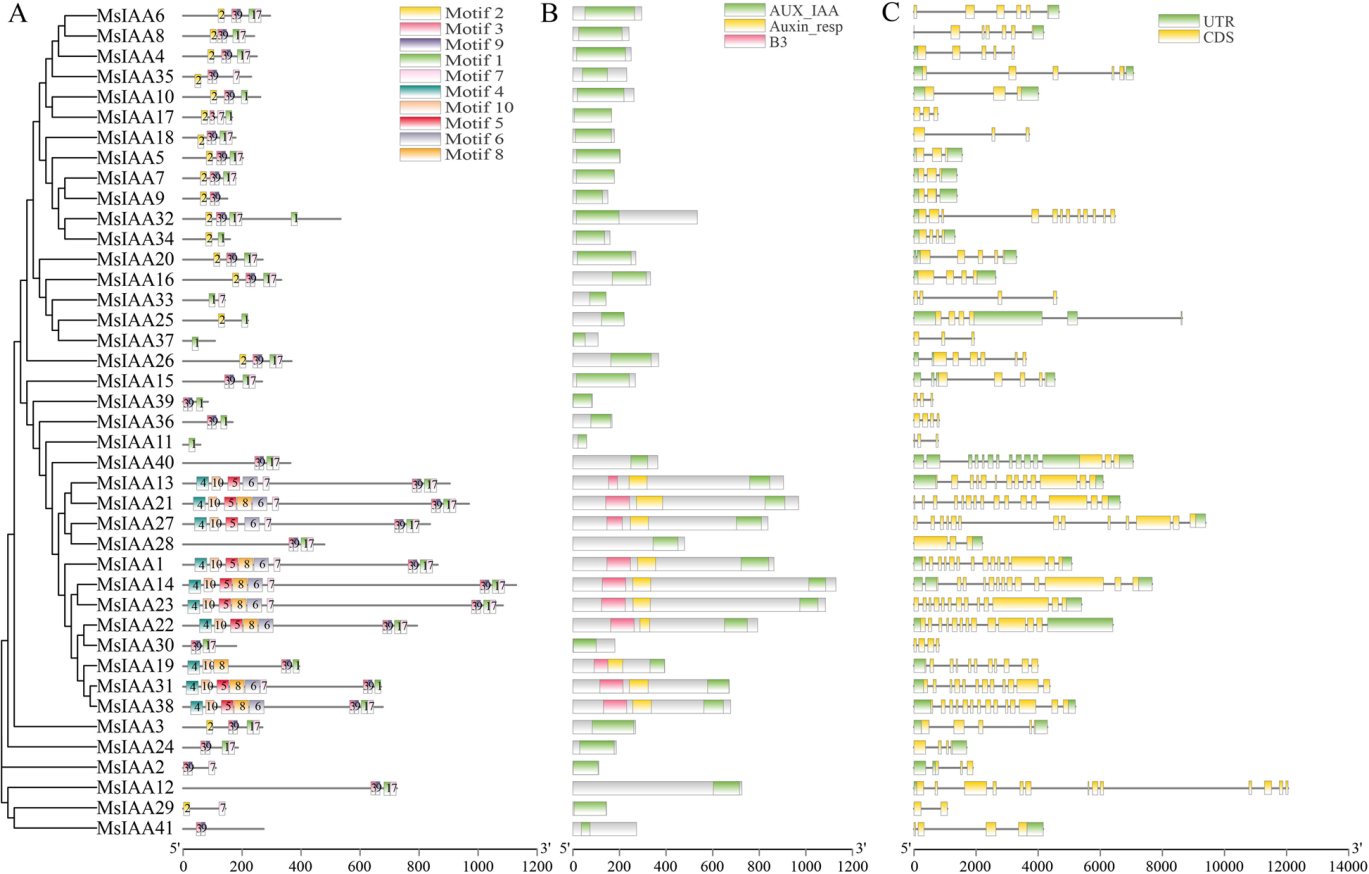
Analysis of conserved motifs and domains and gene structure of MsAux/IAA proteins. **A**: Gene conservative motif analysis; **B**: Protein conservative domain analysis; **C**: Gene structure analysis

MEME tool was used to identify the conserved motifs of the MsIAA proteins, and we obtained visualizations of the 10 conserved motifs. It was showed that aspartic acid residue (D), glycine residue (G), leucine residue (L), serine residue (S), valine residue (V), methionine residue (M), histidine residue (H), alanine residue (A), phenylalanine residue (F), proline residue (P), arginine residue (R), tryptophan residue (W), lysine residue (K), asparagine residue (N), and tyrosine residue (Y) and cysteine residue (C) were extremely conserved (Fig. 4). Further analysis of the distribution of 10 motifs on 41 MsIAA proteins revealed that motif 1, motif 3, motif 7, and motif 9 were highly conserved motifs in the *MsIAA* gene family, appeared in most *MsIAA* gene members, indicating that they were also the four most important motifs in the *MsIAA* gene family (Fig. 5A). On the other hand, we also found that motif 2 was a mainly highly conserved motif in the MsIAA subfamily Class II, III, IV, and V. In addition, motif 4, motif 5, motif 6, motif 8, and motif 10 were mainly highly conserved in the MsIAA subfamily Class VIII.

Analysis of the conserved domains of the MsIAA protein sequence revealed that all MsIAA contain an auxin Aux_IAA structural domain. Interestingly, 10 MsIAAs (MsIAA1, 13, 14, 19, 21, 22, 23, 27, 31, 38) all contain Aux_IAA, B3, and Auxin_resp domain, and they all belong to Class VIII. Combined with the above analysis, we speculated that motif 4, motif 5, motif 6, motif 8 and motif 10 mainly encoded the B3 and Auxin_resp domains (Fig. 5B). To determine the structural differences of the *MsIAA* genes in order to reveal their function, regulation and evolution, the *MsIAA* family numbers were analyzed for gene structure. The results showed that the number of CDS and TR regions in *MsIAA* genes all varied from 2 to 15, and all *MsIAA* genes contained CDS sequence, while 32 *MsIAA*s contained UTR regions and the remaining 9 have no UTR regions. Among the 41 *MsIAA* genes, *MsIAA21* has the most CDS (*n* = 15), followed by *MsIAA1* and *MsIAA38* with 14 CDS, while *MsIAA9* and *MsIAA29* only contain 2 CDS. Through phylogenetic analysis, it was found that the CDS distributions of Class VIII members in Group B of the MsIAA family were the highest (Fig. 5C).

### Chromosomal localization and homology analysis of *MsIAA* gene in alfalfa

**Fig. 6:**
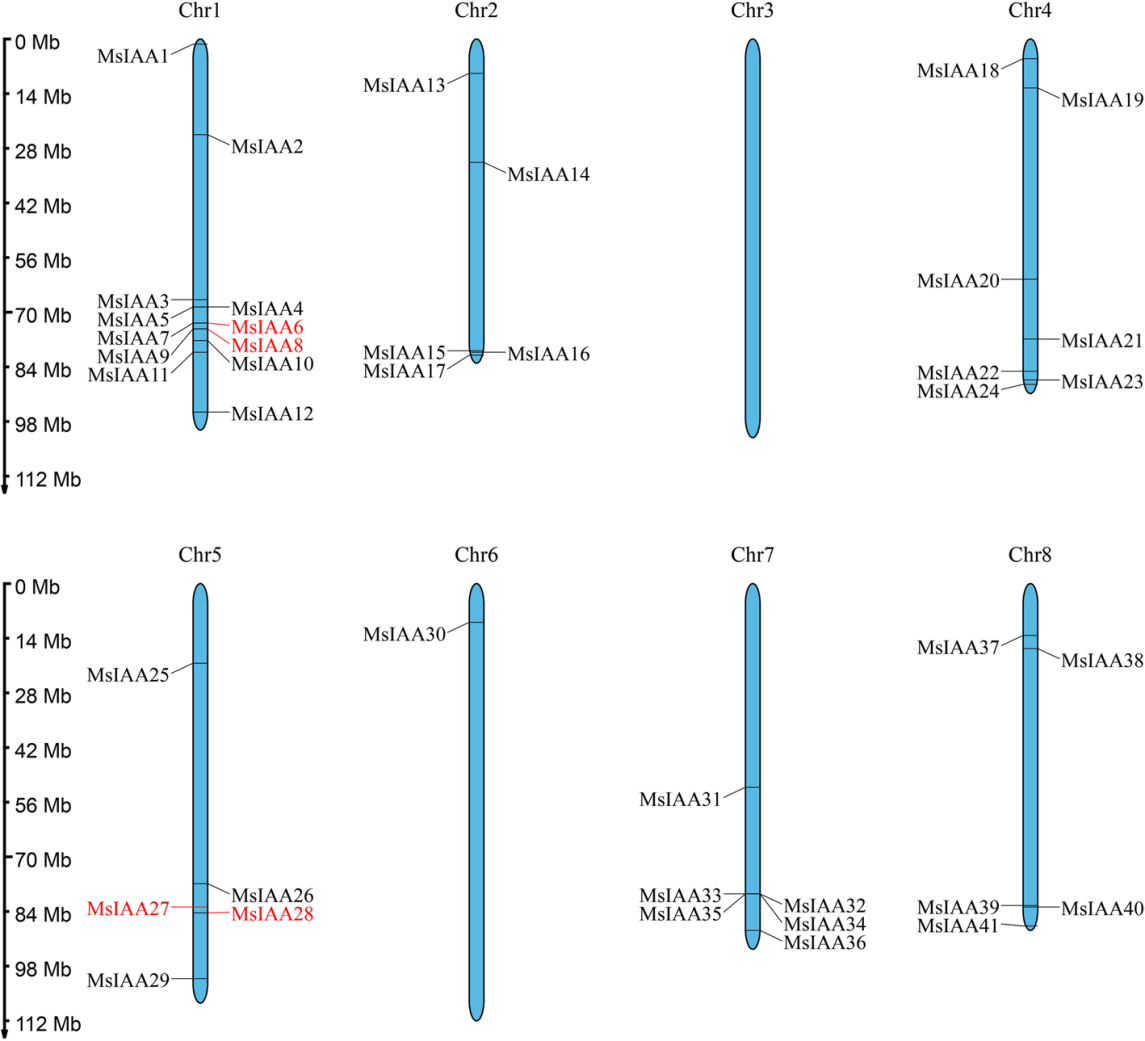
Distribution and location of MsAux/IAA gene on alfalfa chromosomes. Chr 1 to Chr 8 represent the linkage group of "Zhongmu NO.1" alfalfa. Each black line indicates the location of the Aux/IAA gene. The red line represents a tandem duplicated of the Aux/IAA gene

**Fig. 7:**
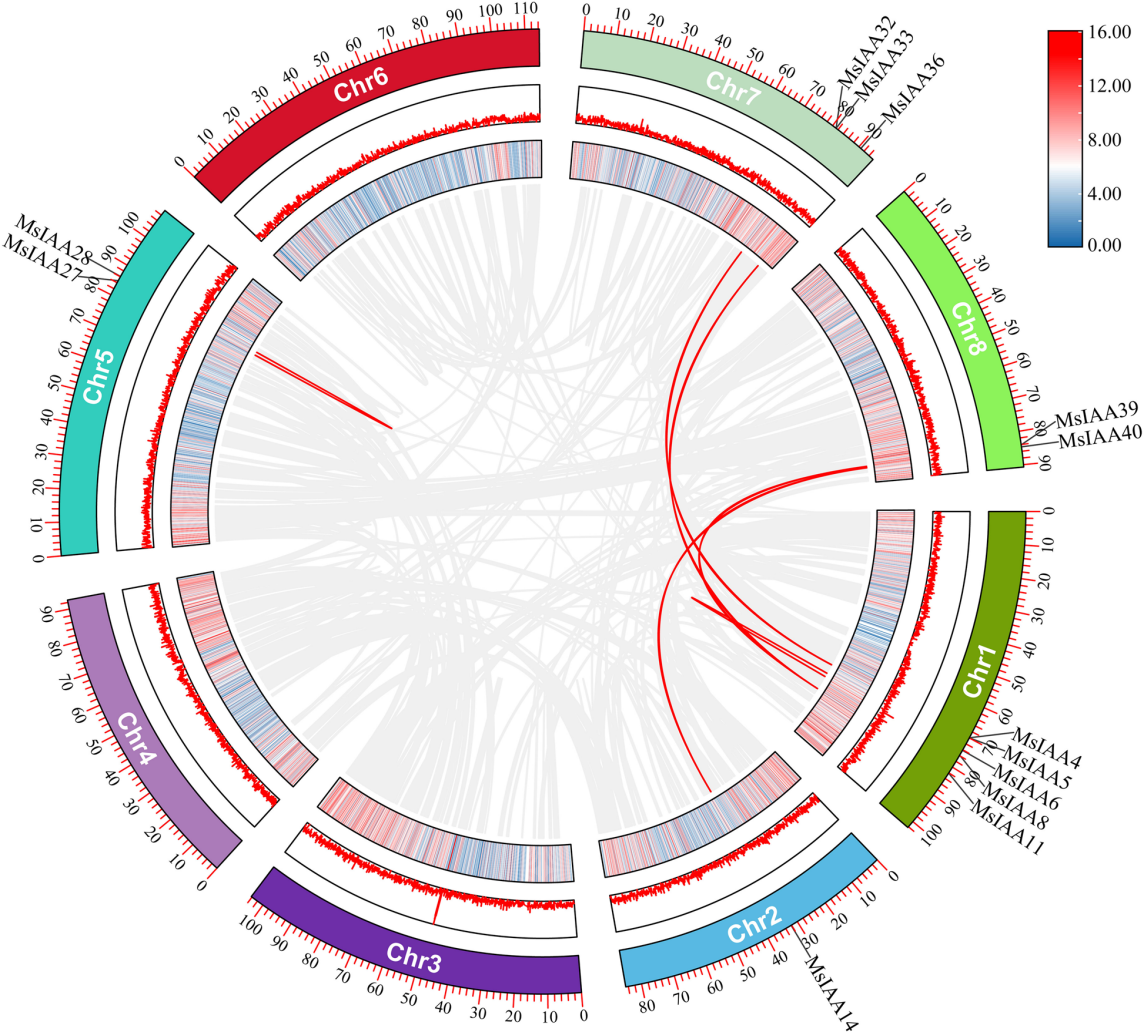
Synteny analysis of the MsAux/IAA gene in the genomes of the *M. sativa*. The gray line represents all the synteny blocks in the alfalfa genome. The green and red lines represent gene pairs and tandem repeat genes in the Aux/IAA gene, respectively. The yellow heat map and the red broken line map represent the gene density and expression level of the MsIAA gene, respectively

**Fig. 8:**
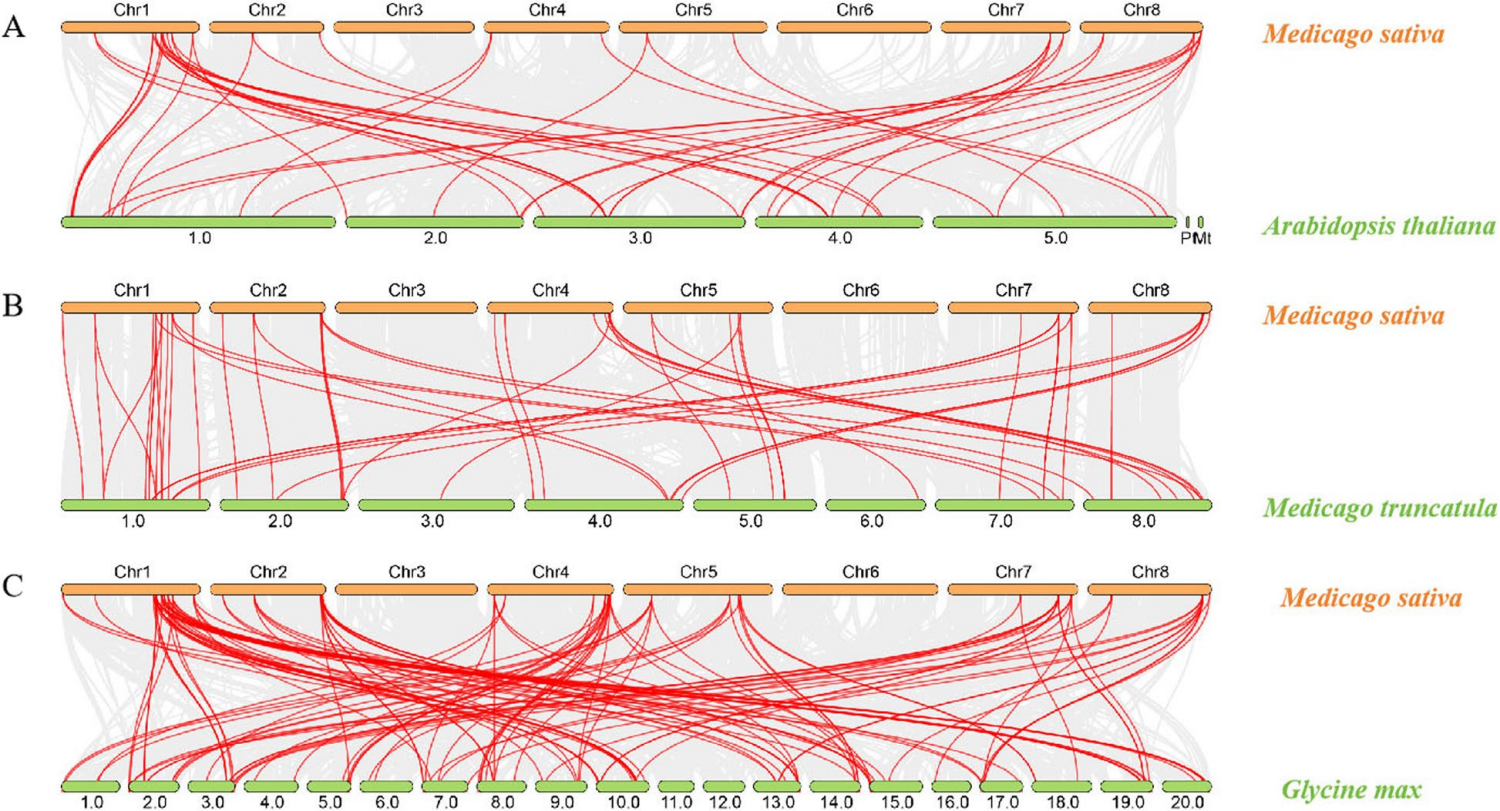
Collinearity analysis between different genomes include *Medicago sativa, Arabidopsis thaliana, Medicago truncatula* and *Glycine max*. **A**: Collinearity analysis between *M. sativa* and *A. thaliana*; **B**: Collinearity analysis between *M. sativa* and *M. truncatula*; **C**: Collinearity analysis between *M. sativa* and *G. max*

To further analyse the collateral homologous gene pair relationship between the *MsIAA* genes in alfalfa, we identified and mapped the chromosomal positions of all identified *MsIAA* numbers (Fig. 6). The results found that 41 *MsIAA* genes were unevenly distributed on seven chromosomes (Chr) in alfalfa, and there was no distribution of *MsIAA* family members on Chr3. And the *MsIAA* genes were the most distributed on Chr1 with 12 members, followed by Chr4, Chr7, Chr2, Chr5 and Chr8, with 7, 6, 5, 5 and 5 members, respectively, while only one member on Chr6. We also performed collinearity analysis to clarify the gene duplication events. The results showed that 5 pairs of segmental duplications and 2 groups of tandem duplications (MsIAA27/MsIAA28 and MsIAA6/MsIAA8) were identified in the *MsIAA* gene (Fig. 7). And 43 pairs of homologous *Aux/IAA*s were identified between *M. sativa* and *A. thaliana* (Fig. 8A), and 50 pairs of homologous *Aux/IAA*s were identified between *M. sativa* and *M. truncatula* (Fig. 8B), while 127 pairs of orthologous *Aux/IAA*s were identified between *M. sativa* and soybean (Fig. 8C). Five *MsIAA*s (*MsIAA2*, *MsIAA5*, *MsIAA6*, *MsIAA8* and *MsIAA32*) had one homologous gene in *A. thaliana*, and fore *MsIAA*s (*MsIAA4*, *MsIAA6*, *MsIAA8* and *MsIAA33*) had one homologous gene in soybean. To determine whether there was selection pressure acting on the *IAA* family genes, we further calculated the Ka/Ks values for *MsIAA* numbers. The results found that the Ka/Ks values of 5 pairs of segmental duplications and 2 groups of tandem duplications were less than 1 respectively, indicating they exert purification of selection effects (x S4).

### Analysis of Cis-regulatory elements of the *MsIAA* gene family

**Fig. 9:**
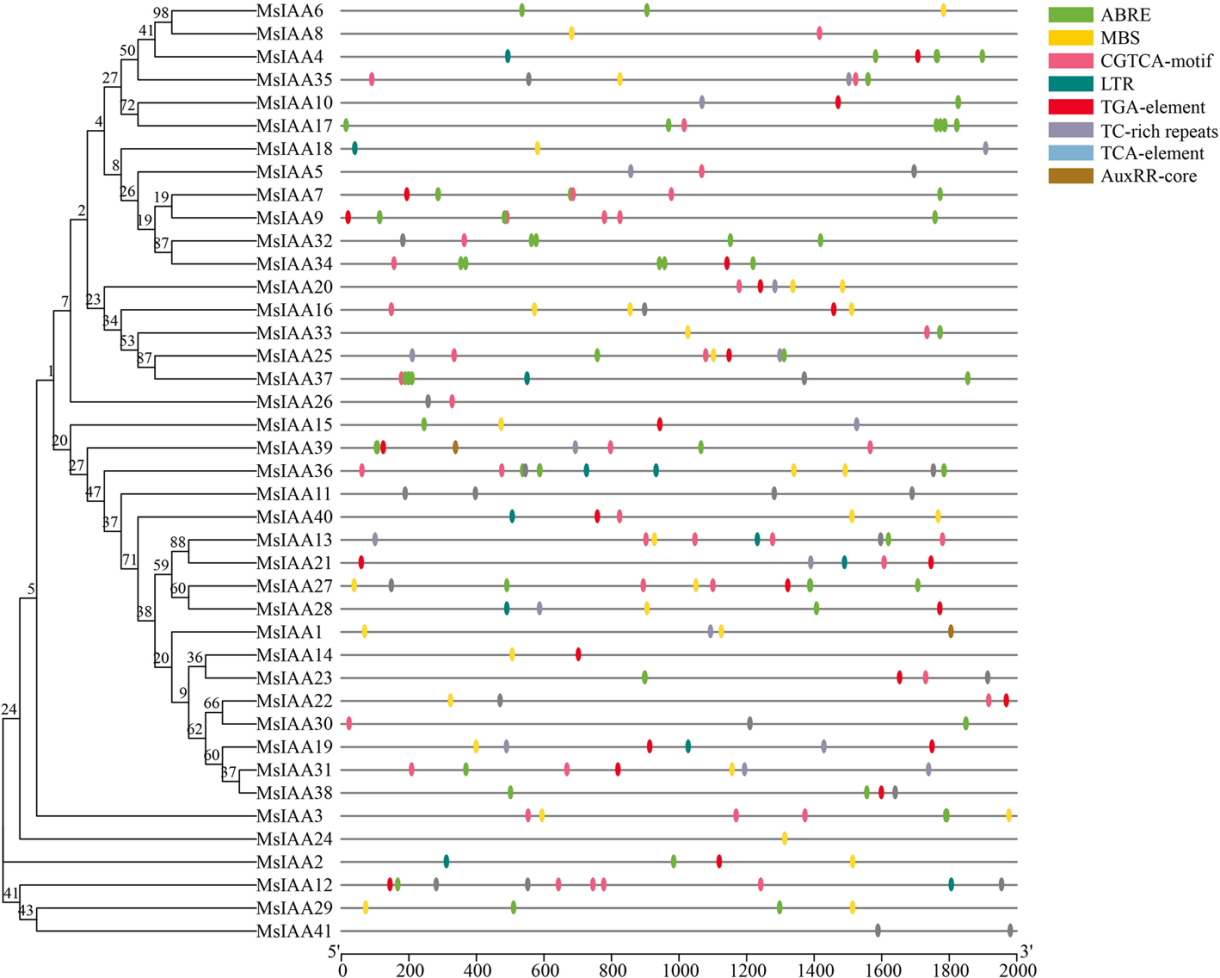
Analysis of Cis regulatory elements in the upstream promoter of MsAux/IAA gene

The cis-regulatory element regulated the expression of genes involved in the stress response by binding to transcription factors, which was located in a specific DNA sequence upstream of the coding sequence of the gene. In this study, ABRE had the most cis-regulatory elements, with 74, followed by CGTCA-motif (*n* = 43), MBS (*n* = 32), TGA-element (*n* = 24), TCA-element (*n* = 23), TC-rich repeats (*n* = 17), LTR (*n* = 12), and AuxRR core (*n* = 2) (Fig. 9). Interestingly, we found that the 2000 bp upstream sequence of the *MsIAA41* gene and *MsIAA11* only contained two and four TC-rich repeats cis-regulatory elements, respectively. Except for TC-rich repeats, there are no other cis-regulatory elements at *MsIAA41* and *MsIAA11*. And the *MsIAA24* gene only contained a MBS elements.

### Protein–protein interaction (PPI) network analysis

**Fig. 10:**
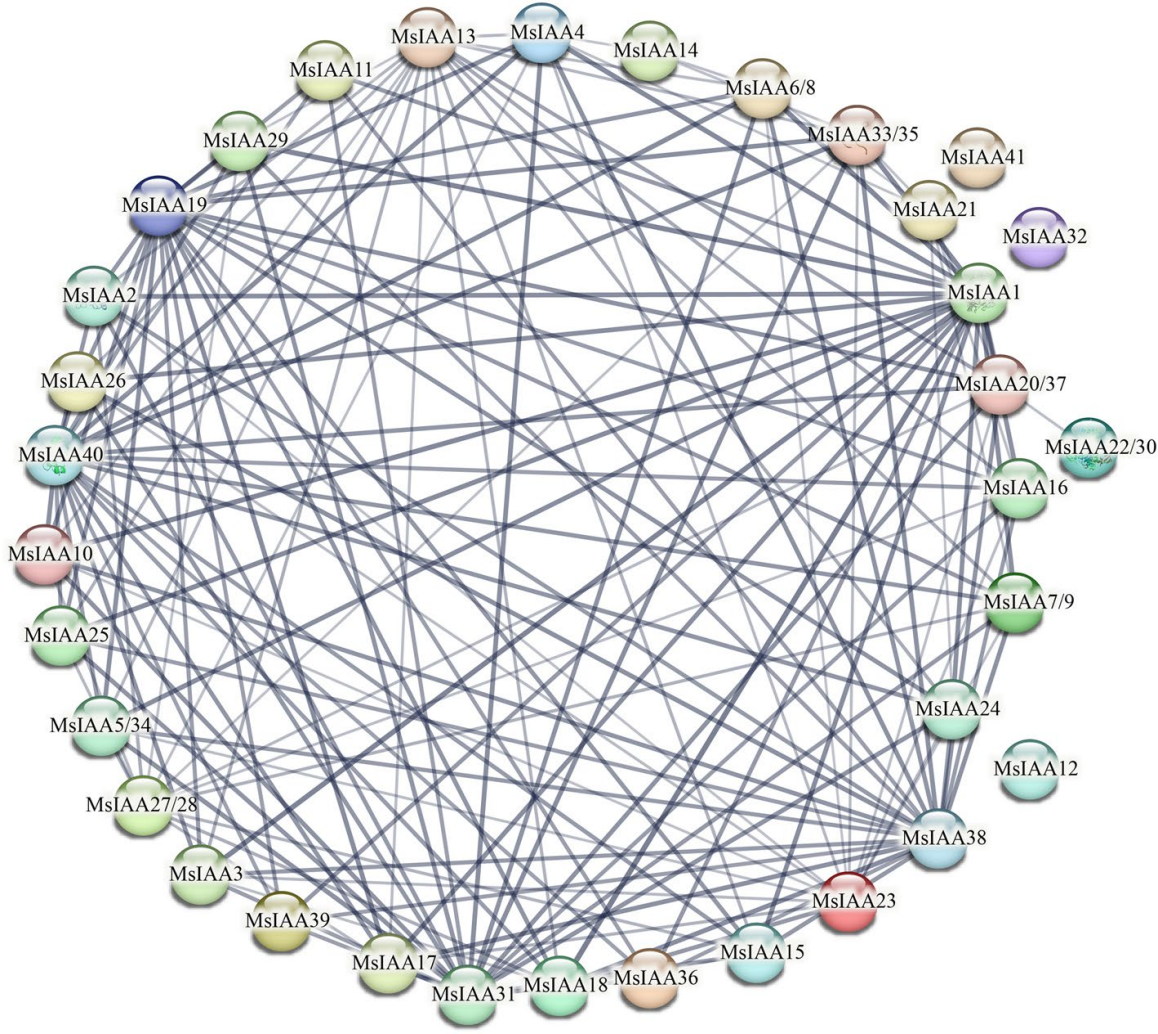
The protein–protein interaction (PPI) network of MsAux/IAA genes and related hub genes

To effectively identify and visualize potential interactions, we analyzed PPIs for 41 MsIAA proteins that can explain the hub gene of this interaction (Fig. 10, Table S5). It was found that 7 MsIAA were repeatedly annotated, such as MsIAA5/34 (3880.B7FMP7), MsIAA6/8 (3880.A0A072VMX0), MsIAA7/9 (3880.G7I486), MsIAA33/35 (3880.I3SRI4), IAA20/37 (3880.A0A072UBZ5), MsIAA22/30 (3880.G7LIT1), MsIAA27/28 (3880.G7KFN6), respectively. And within the hub gene network, *MsIAA38* (3880.A0A072TM91), *MsIAA21* (3880.A0A072TTL5) and *MsIAA31* (3880.A0A072U1C1) had the highest number of interactions, suggesting that these genes played a crucial role in regulating the function of the *IAA* gene family. In addition, three of the hub genes, *MsIAA12*, *MsIAA32* and *MsIAA41*, had yet to be annotated and required further investigation.

### Analysis of the expression patterns of *MsIAA* gene family numbers under different treatment

**Fig. 11:**
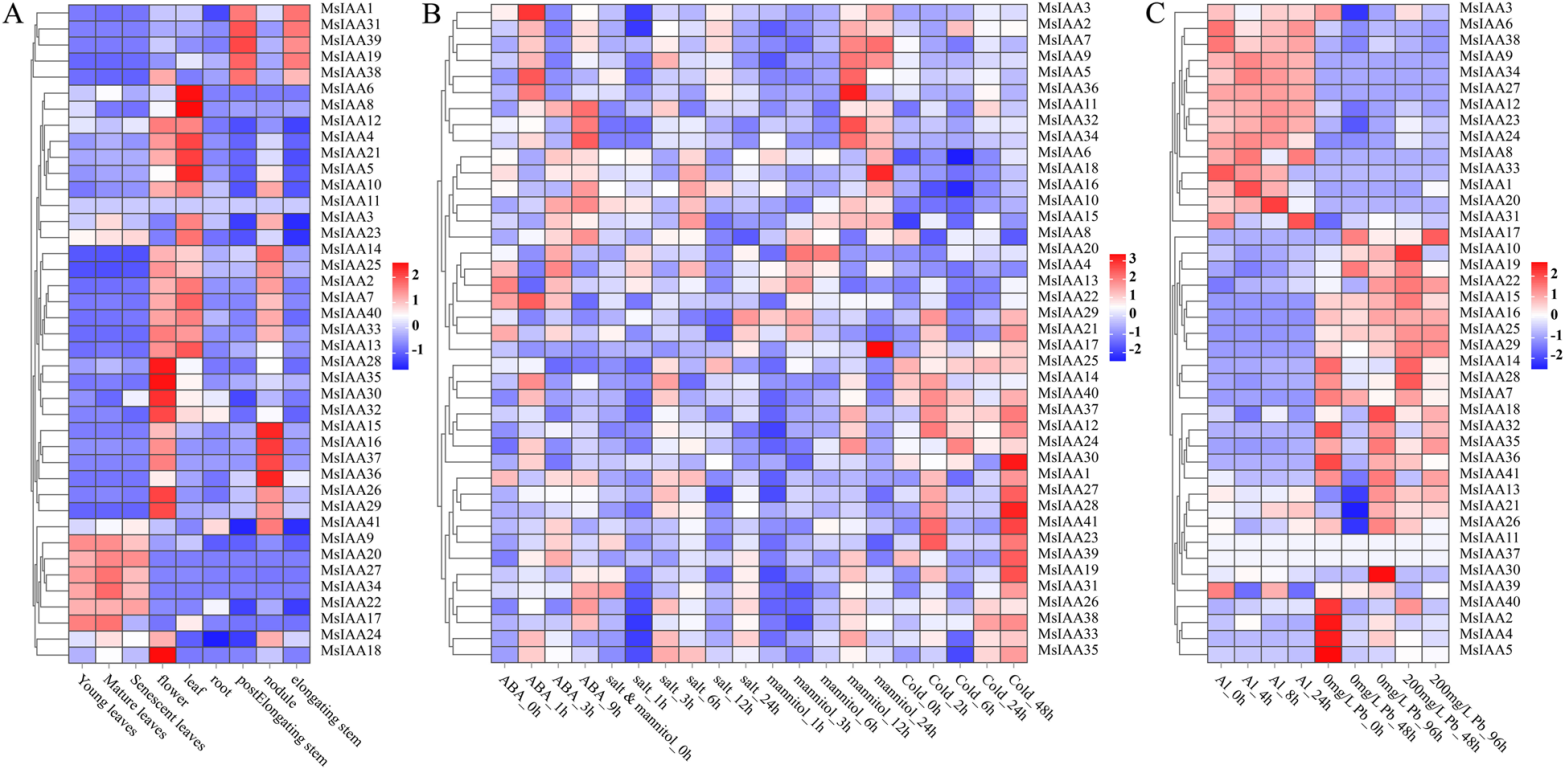
Analysis of the expression pattern of the MsAux/IAA gene family. **A**. Tissue specific expression pattern analysis; **B**. Analysis of expression patterns under salt, drought, and cold stresses; **C**. Analysis of expression patterns under Al and Pb stress

*Aux/IAA*s, as repressors that respond to auxin and regulate its gene expression, play an important role in auxin signal transduction, thereby regulating plant growth and development, such as tissue differentiation and response to abiotic stress. Therefore, this study utilized the alfalfa database to obtain 41 expression patterns of *MsIAAs*, mainly divided into three types, including specific expression in different tissues (young leaves, natural leaves, sensory leaves, flowers, leaf, root, post elongating stem, node and elongating stem), common abiotic stress (ABA, salt, dry and cold), and heavy metal stress (Al and Pb) (Fig. 11). The tissue-specific expression of *MsIAAs* were related to its specific function in specific tissues, and the expression patterns of 41 *MsIAAs* in 8 different tissues were divided into 4 types (Fig. 11A). Under ABA, salt, drought, and cold stress, the expression of *MsIAAs* were very diverse. Our analysis showed that the expression of *MsIAA* under different stress conditions was not only related to the type of stress, but also limited by the duration of stress, which is closely related to MsIAAs being short-lived proteins (Fig. 11B). In addition, we also found that under heavy metal stress, except for the type and duration of stress, the expression of *MsIAAs* were also related to stress concentration, indicating that *MsIAA* have very complex regulatory mechanisms under abiotic stress (Fig. 11C).

**Fig. 12:**
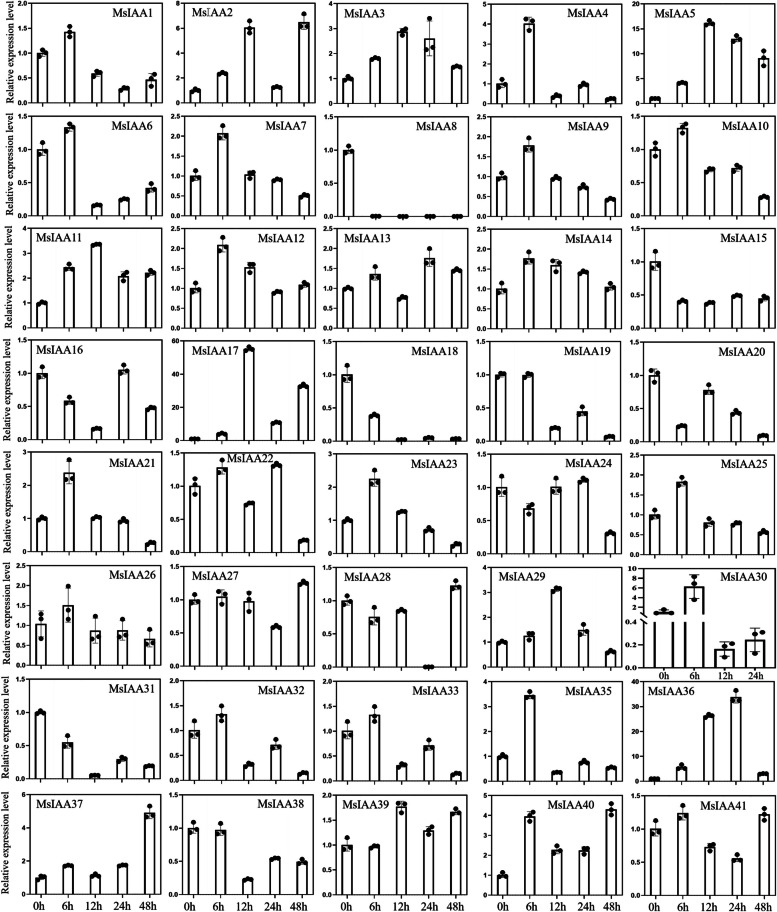
The expression pattern analysis of Aux/IAA genes under drought stress treatment for 0, 6, 12, 24 h and 48 h using qRT-PCR. Data are presented as the mean ± standard deviation

To reveal the possible function of *MsAux/IAA* gene family numbers in response to plant drought stress, we analyzed the expression pattern of the 41 *MsIAA* genes identified in root under the drought stress (Fig. 12). The result found that *MsIAA34* showed no or very low expression and was not further analyzed, and *MsIAA30* was no expression at 48 h. Among others, 13 *MsIAA* genes (*MsIAA1/4/6/7/9/10/21/23/25/26/32/33/35*) were significantly upregulated at 6 h, and the expression level decreased in the following time including 12, 24 and 48 h. Interestingly, *MsIAA30* also belonged to this type but lack of 48 h. And 9 *MsIAA* genes (*MsIAA3/5/11/14/17/36/37/39/40*) were upregulated under drought stress at 6, 12, 24 and 48 h when compared with CK. On the contrary, 9 *MsIAA* genes (*MsIAA8/15/16/18/19/20/24/31/38*) were downregulated under drought stress at 6, 12, 24 and 48 h when compared with the CK. The expression patterns of the remaining eight genes were period-specific, with *MsIAA2* upregulated at 12 and 48 h, *MsIAA12* and *MsIAA22* upregulated at 6 and 12 h, *MsIAA13* only downregulated at 12 h, *MsIAA27* and *MsIAA28* significantly low expression at 24 h, *MsIAA29* only significantly high expression at 12 h, as well as *MsIAA41* upregulated at 6 and 48 h and downregulated at 12 and 24 h.

## Discussion

Significant progress has been made in regulating the function of *IAA* genes in auxin signaling in understanding auxin sensing and signaling [30]. However, much remains unknown regarding the processes of *Aux/IAA* involvement in the auxin signaling pathway. At present, research on *Aux/IAA* in the auxin signaling pathway mainly focuses on various aspects of plant growth and development, but little attention has been paid to the role of *Aux/IAA* in the response to auxin mediated environmental interactions [41]. In this study, we performed the genome-wide identification and related analysis of *Aux/IAA* genes in alfalfa using the whole genome of tetraploid cultivated alfalfa, and analyzed the expression pattern of 41 *MsIAA* genes under 15% PEG simulation drought by qRT-PCR, which is important to reveal the possible function of *Aux/IAA* in plants in response to drought stress.

### Various protein physicochemical, peptide structure, secondary and tertiary structure analysis reveal functional diversity of the MsIAA proteins

With the increasing popularity of whole genome sequencing technology, the *Aux/IAA* genes of several species, including most crops, have been identified. The number of *Aux/IAA* genes varied significantly between different species, such as only one in *Marchantia polymorpha* [42], 28 *Aux/IAA* genes in *Arabidopsis* [43], 25 in *M. truncatula* [9], 63 in soybean [44], and 119 in *Brassica napus* [45]. In this study, 41 *IAA* genes were identified in alfalfa with higher numbers than some leguminous plants including *M. truncatula*, which may be related to the fact that alfalfa is the main tetraploid cultivated specie, and also indicated that alfalfa *Aux/IAA* gene was not overextended in the evolution. To further illustrate the function of *MsIAA* genes, physicochemical properties analysis of encoding protein were performed, and it was found that 41 MsIAA proteins had various physicochemical characteristics. The number of amino acids of most of *MsAux/IAA* (26) were less than 7, the average value was 6.66; the coefficient of instability of *MsAux/IAA* (26) was greater than 40, the average value was 46.19, mostly unstable proteins; the average value of aliphatic amino acid index was 74.84, the average value of protein hydrophobicity coefficient was -0.45, most of which were hydrophilic proteins. It can be seen that although there were obvious differences in the specific values, the performance classification of physicochemical properties was relatively single, reflecting the coexistence of functional diversity and unity of *Aux/IAA* genes, suggesting that different *MsIAA* genes might function in different microenvironments. Subcellular localization indicated that most of the proteins were localized to the nucleus (25), indicating that the nuclear localization signal in Aux/IAA proteins was conserved [46]. And they did not have any signal peptide, guide peptide, or transmembrane structure, indicating that the *Aux/IAA* gene in alfalfa did not act as a transcriptional repressor for cell secretion or transport, but only acted in the nucleus. Among them, three genes (*MsIAA4/9/8*) located on chloroplast had a typical transmembrane domain, and *MsIAA29* had two typical transmembrane domains, indicating that these four genes on chloroplast not only functioned in chloroplast, but also secreted and transported, and then played a role in other organelles. These characteristic properties of alfalfa *Aux/IAA* were similar to the *Aux/IAA* features in most plants [46], which may be related to the conservatism of *Aux/IAA*, implying their similar functions.

Gene structure can intuitively determine gene function, therefore, in order to more fully reveal the function of *MsIAA*, we further analyzed the secondary and tertiary structure of 41 genes. It was found that the secondary structure of the majority of the largest proportion were α-coil and irregular coil. At the same time, we predicted the tertiary structure of all *MsIAA* genes, the results showed that each gene contained a helix-fold-helix structure, suggesting that the helix-fold-helix structure was the essential spatial structure of the *MsIAA* gene. The acquisition of physicochemical properties, secondary and tertiary structure can provide clues for further studying the function and interaction of proteins, and it was an indispensable component in the analysis of plant gene families. The wide variability in physicochemical properties among the members of the *MsIAA* gene family in this study suggested that different MsIAAs proteins may function together and participate in regulating these processes under variable microenvironmental conditions including plant growth and development, as well as different biological and abiotic stresses.

### The phylogenetic relationship and domain analysis demonstrates specificity of *MsIAA* gene function

The elucidation of evolutionary relationships and the prediction of the potential function of genes can be achieved through phylogenetic analysis [47]. Earlier studies showed that the evolutionary relationship of *Aux/IAA* genes can be divided into five major clades [48]. There were also studies that classified *Aux/IAA* genes into two major groups [44], A and B similar to *Arabidopsis* [43] and rice[49], which were further subdivided into 10 subgroups. Analysis of the evolutionary tree constructed in this study made it more reasonable to adopt the second classification method. The evolutionary analysis showed that I-V in class A all contained *MsIAA* genes, while class IX in group B did not contain *MsIAA* genes, indicating that the divergence formation of MsIAA transcription repressor family members may be later than the species differentiation in alfalfa.

Wu *et. al* [48]*.* had reported that altered protein properties were determined by the absence of domains and relevant scholars have elucidated the function of the domain in Aux/IAA proteins. This study used MEME to identify 10 conserved motifs of MsIAA, the result found that D, G, L, S, V, M, H, A, F, P, R, W, K, N, Y and C residue were extremely conserved, which was similar to the results obtained in most plants [30]. The *MsIAA* gene domain is the core of the Aux/IAA transcription factor, which can activate the downstream genes by interacting with the promoters of the downstream genes, thereby enabling them to function. Further analysis of the motif distribution revealed that motif1, motif3, motif7, and motif9 were highly conserved in MsIAA proteins, corresponding to domain I-domain IV. These genes were classified into typical *Aux/IAA* (26), and the remaining 15 members belong to atypical *Aux/IAA* based on their domain structure, or called atypical members [21], and motif4, 5, 6, 8, and 10 were mainly found in the class VIII. These atypical *Aux/IAA* genes mainly lack domain I (EAR-motif), suggesting that these members may participate in other biological processes that do not require ARF-mediated auxin regulation [3]. The atypical Aux/IAA proteins played key roles in plant growth and development processes as well as in their adaptation to various environmental conditions, but their specific functions remained unknown [45], which indicated that atypical auxin signaling pathways may be involved in growth and development in *M. sativa* [50]. These atypical Aux/IAA proteins were more long-lived than typical proteins [51]. It had been shown that this atypical Aux/IAA protein may have special functions in plant auxin response processes and signal transduction [21]. Previous studies had reported that the C-terminus was conserved, and the N termini of Aux/IAA proteins interact with ubiquitin ligase receptors through motif-based interactions [52]. In this study, the C-terminus was composed of motif 1, 2, 3, and 4, and the N-terminus consisted of motif 1, 7 and 9, showing that two domain motif 1 and 3 were conserved in MsIAA, and two domain additional motif 7 and 9 played a major role. In this study, only 21 MsIAAs had four complete domains, implied some variability in the function among the MsIAAs members.

### The gene duplication analysis shows that *MsAux/IAA* genes tend to lose mutations by purifying selection

Gene duplication events determined the diversification and specificity of gene function, which often occurred between members of the same gene family during plant evolution [53, 54]. Previous studies have found that more gene duplication events in plants are segmental tandem duplication, which acts to expand gene families [55]. In this study, only two pairs of tandemly duplicated genes of the *MslAA* gene family were detected, namely *MsIAA27/MsIAA28* and *MsIAA6/MsIAA8*. And we identified five pairs of segmental duplicated genes, suggesting it played a major role in the amplification of the *Aux/IAA* gene family in alfalfa. Furthermore, the Ka/Ks ratios were all less than 1, suggesting that *MslAAs* were under purifying selection, and indicating that *M. sativa* had undergone local gene duplication and shares an ancient round of gene duplication with other leguminous plants [56], or which indicated that all gene pairs evolved mainly under the influence of purification selection pressure, and the functional differences after fragment replication were limited [44]. These results were consistent with previous study [57], which reported that *Aux/IAA* genes tend to lose mutations by purifying selection and subsequently adapt to current microenvironmental conditions.

### Cis-acting element analysis demonstrates that *MsIAA* genes regulates the response to abiotic stress in alfalfa

Correlation studies suggested that temporal and spatial changes in gene expression were influenced by promoters, and regulation of gene function was determined by cis-elements within the promoter through interactions with trans-acting factors [58]. Multiple promoter elements associated with abiotic stress and hormones had been identified in the *Aux/IAA* genes [9]. The analysis of *MslAAs* promoter found seven major cis-elements that had specific functions, including drought-inducibility (MBS), defense and stress responsiveness (TC-rich repeats), hormone responsive elements (AuxRR-core, ABRE, TGA-element, CGTCA-motif), low-temperature responsiveness (LTR), and it was found that *MsIAAs* may respond to various stimuli, including auxin, GA, ABA, salicylic acid, JA, drought, salt, heat stress, and low temperature, indicating that these genes were involved in phytohormone signaling and/ or stress response. Many *MsIAA* genes contained the AuxRE motifs that bind to the downstream *ARF* in their promoters, which was important for the transcriptional activation in auxin signaling [59]. The presence of these cis-acting elements explained the universality of *MsIAAs* function as well as the specificity of tissue expression. Some promoter elements were enriched multiple times in the promoter regions of *MsIAA* genes. Most genes had two or more genetic components, and only three genes each contained one class of cis-elements, *MsIAA41* and *MsIAA11* gene only contained two and four TC-rich repeats cis-regulatory elements, and *MsIAA24* gene only contained a MBS elements. Taken together, multiple cis-regulatory elements exist in the upstream region of the *MsAux/IAA* genes. These results provide new evidence that support for further studies on the involvement of *Aux/IAA* genes in plant growth and development processes and in abiotic stress responses.

Many physiological processes including the regulation of signal transduction and gene expression involve protein–protein interactions of plant. Auxin responses were mediated by interaction between *Aux/IAA* genes and other genes [12]. Thus, studying the interaction of Aux/IAA proteins in *M. sativa* was significant. In this study, we found that the proteins encoded by *MsIAA* genes formed a complex regulatory network, among which, three genes (*MsIAA38/21/31*) were at the core, and three genes (*MsIAA12/32/41*) acted independently and had no interaction with other *MsIAA* genes. Moreover, there were seven duplicate genes in alfalfa compared to the *M. truncatula*, which may depend on the genome size of both species [39, 60].

### The expression analysis of *MsIAA* genes in tissue development, abiotic stress responses, and their potential functions under drought stress

The underlying function of a gene influences its temporal and spatial expression. Several *IAA* genes showed specific and overlapping expression patterns in different tissues and developmental periods in alfalfa, suggesting that they regulated the initiation and development of different organs [9]. The expression patterns of *Aux/IAA* genes in turnip (*Brassica rapa*) and pepper (*Piper nigrum*) were tissue-dependent [61, 62]. The *MsIAA* genes also exhibited tissue-specific transcriptional patterns, indicating their important roles in leaf, root, stem or flower development in the study. In addition, we used previous transcriptome data to obtain the expression patterns of 41 *MsIAA* genes under ABA, salt, mannitol, cold, Al and Pb treatment and showed that different genes respond differently to abiotic stress. The expression patterns generated by the promoter activity and/or the molecular properties of the gene product determine the similarities and differences of the *Aux/IAA* genes [63]. Overall, the expression of several *Aux/IAA* genes in different tissues and developmental periods in alfalfa indicated that they participated in the developmental process of specific tissues.

Promoter sequence analysis of *MsIAAs* revealed the presence of drought-responsive cis-regulatory elements, hence further investigation of its role under drought stress response/signaling was valuable. The quantitative real time polymerase chain reaction (qRT-PCR) is a useful and convenient tool for reflecting the transcript levels of target genes under any condition [64]. More and more evidence suggested that *Aux/IAA* genes were responsive to drought stress [41]. Therefore, the expression patterns of the 41 *AUX/IAA* genes using qRT-PCR were analyzed under drought stress. We found that the 41 *MsIAA* genes were divided into five categories according to their expression patterns, and in the first category, *MsIAA34* was not expressed under drought stress. Fourteen *MsIAA* genes in the second category were upregulated only at 6 h, and these genes responded rapidly to short drought stress, consistent with the characteristics of *MsIAA* as a short-lived gene [11]. Nine *MsIAA* genes in the third category were up-regulated, and nine genes in the fourth category were downregulated under drought stress. The expression of eight genes in the fifth category was period-specific under drought stress. Previous studies had found that *MtIAA* genes showed extensive responses to drought stresses, and many *SbIAA* genes of *Sorghum bicolor* had been found down-regulated under drought conditions [9, 65]. Similarly, many *Aux/IAA* genes in other plant species were induced by drought treatment [34]. *Aux/IAA* genes were believed to play a negative regulatory role in response to drought stress. For instance, under drought treatment, the expression level of *IAA4* in grape (*Vitis vinifera*) decreased [66]. However, both *OsIAA6* and *OsIAA20* were upregulated during drought stress and the over-expression lines showed enhanced drought resistance, indicating that *OsIAA6* and *OsIAA20* were positive regulators of drought stress [35].

It is known that *Aux/IAA* genes mainly binds to ARF to regulate plant endogenous auxin concentration and then in response to drought stress. Furthermore, *Aux/IAA* genes can also be integrated with other signal transduction pathways in response to the external environment or intrinsic genetic signals. Previous study has found that *IAA5*, *IAA*6 and *IAA19* can be directly regulated by DREB transcription factors (DREB2A and DREB2B) and play an important role in drought response [67]. Further studies showed that *IAA5*, *IAA6* and *IAA19* regulated plant drought tolerance by regulating glucosinolate levels [31]. Recent study has found that under drought conditions, the interaction between *ZmCCT* and *ZmAux/IAA8* regulates the expression of downstream important drought induced genes [68]. These studies all indicate that *Aux/IAA* genes can jointly regulate plant drought stress by combining with other pathways to form regulatory modules or expression networks. In this study, nine genes in the third category and nine genes in the fourth category had opposite expression patterns, indicating that *Aux/IAA* can regulate drought stress in different ways, and the mechanism is also more complex. Thus, information on the *Aux/IAA* gene family in alfalfa and the functional characteristics of the *MsIAA* genes under drought stress remains limited. In the future, the functions of *MsIAA* genes in the drought stress of alfalfa should be further investigated using transgenic or gene-editing techniques.

## Conclusion

In this study, we identified 41 *MsIAA* genes across the genome of *M. sativa* and analyzed gene structure, protein features, and promoter sequences, and explored their potential roles in regulating drought response. With bioinformatics analysis, we observed that the *MsIAAs* were highly conserved and can be divided into 10 classes based on phylogenetic analysis. At the same time, the structure of MsIAA protein was also different, indicating that the properties and functions of the protein had changed. Moreover, hormone responsive and abiotic stress-related elements were identified in the promoter of *MsIAA* genes. Segmental duplication was the main mode of gene number amplification of the *Aux/IAA* gene family in *M. sativa*. The expression of *Aux/IAA* genes showed tissue specificity in root, stem and leaf development of alfalfa, and *MsIAA* can regulate abiotic stress in plants. Analysis of expression patterns under drought stress revealed that *MsIAA* had a broad response to drought stress, which can serve as a new perspective on improving the drought resistance of alfalfa. In general, *MsIAAs* are essential to plant development, auxin response, and abiotic stress responses in *M. sativa*. Our results are important for further studies on the involvement of auxin under drought stress and contributes additional evidence that will improve future genome annotations for this crop.

## Materials and methods

### Experimental material and treatment of drought stress

The material used in this experiment was "Zhongmu No.1" alfalfa. Alfalfa seeds with full and single shape were selected for surface sterilization, planted in pots with vermiculite: nutrient soil = 1:1 (20 cm diameter, 20 cm high), and five biological replicates were set. They were grown in a culture room with a 16 h light/8 h dark cycle, relative humidity of 60% and temperature of 22℃. After 15 days, drought treatment (15% PEG-6000 solution) was performed, each seedling to be treated was irrigated (until fully thoroughly), and roots were collected at different stress time points (6, 12 24 and 48 h). Untreated seedling (0 h) was used as a control (CK). All roots were frozen with liquid nitrogen and stored at -80℃.

### Identification of the *MsIAA* gene family and the physicochemical properties analysis

The alfalfa genome data, nucleic acid and protein sequences were downloaded from the online website (https://figshare.com/projects/whole_genome_sequencing_and_ assembly_of_Medicago_sativa/66380) and used for local database construction [39]. The Hidden Markov Model (HMM) profile of Aux/IAA (PF02309) was downloaded from the Pfam database (http://pfam.xfam.org/) and further filtered using HMMER 3.0 software, with an E-value threshold of 1.0 and the remaining parameters by default. Redundancy was removed using the Expasy online database (https://web.expasy.org/decrease_redundancy) [69]. To verify the accuracy of the MsIAA protein sequence after removing redundancy, Aux_IAA domain of the MsIAA protein sequence was further identified on the Pfam website (http://pfam.xfam.org/search#tabview = tab1) and NCBI-CDD Search (https://www.ncbi.nlm.nih.gov/Structure /cdd/wrpsb.cgi), and sequences without the Aux_IAA domain were removed [70]. And the predicted genes are sequentially named MsIAA1 ~ MsIAA41 according to their order of appearance in the genome. Preliminary prediction of physicochemical properties of 41 identified MsIAA protein sequences, including the number of amino acids, molecular weight, theoretical isoelectric point, instability index, aliphatic index, and grand average of hydropathicity were performed by the online network ProtParam (https://web.expasy.org/protparam/) tool. The subcellular localization of all MsIAA genes was predicted by the online tool WoLF PSORT (https://www.genscript.com/wolf psort.html). Signal peptides were analyzed by SignalP 5.0 (http://www.cbs.dtu.dk/services/SignalP-5.0) and transmembrane structures using TMHMM 2.0 (http://www.cbs.dtu.dk/services/TMHMM2.0). And leading peptides prediction were analyzed using Target P-2.0 (http://www.cbs.dtu.dk/services/Target P-2.0). Addition to, secondary structures were predicted using SOPMA (https://npsa-prabi.ibcp.fr/cgi-bin/npsa_automat. plpage = npsa_sopma.html). Tertiary structure were predicted by SWISS-MODEL (http: //swissmodel.expasy.org/interactive).

### Phylogenetic analysis, gene structure, and motif composition of MsIAA proteins

To analyze the phylogenetic relationship of MsIAA proteins, multiple sequence alignment of MsIAA, AtIAA (*Arabidopsis thaliana*), MtIAA (*Medicago truncatula*) and GmIAA (*Glycine max*) protein sequences was performed using MEGA 7.0 software and automatically trimmed with TrimAL [71]. Phylogenetic tree was constructed by maximum-neighbor-Joining (Neighbor-Joining, NJ) method using MEGA 7.0 software. Referring to the classification of IAA of *Arabidopsis* and soybean, the *MsIAA* family genes were classified into Class I—X [44]. The motifs of all 41 MsIAA proteins analyzed by MEME 4.12.0 online tool (http://meme-suite.org/) and their type and order, and the motif features were visualized by TBtools [72]. The parameter settings for MEME is that the maximum number of motifs is 10.

### Analysis of chromosome mapping and gene duplication

The CDS sequences and gene sequences corresponding to all *MsIAA* genes were obtained from the alfalfa genome file, and the intron and exon structures of *MsIAA* genes were predicted by using the GSDS 2.0 (gene structure display server) (http://gsds.gao-lab.org/) online website. In addition, to explore the distribution characteristics of *Aux_IAA* genes on chromosomes, MapGene2Chrome V2.0 (http://mg2c.iask.in/mg2c_v2.0/) was used to map the chromosome location based on the GFF3 data from alfalfa genome annotation file. Localization of genes on the chromosome and collinearity analysis of gene duplication within the *M. sativa* and between different species was completed using TBtools.

### Cis-regulatory element analysis

The cis-regulatory elements in the 2000 bp sequence upstream of the transcription start site of the *MsIAA* gene were identified by the PlantCARE database (http://bioinformatics.psb.ugent.be/webtools/plantcare/html/), which are binding sites for transcription factors and regulate the precise initiation and transcription efficiency of gene transcription by binding to transcription factors.

### Heatmap analysis of MsIAA gene response to abiotic stress and tissue specificity

Heatmap of the expression profile data of *MsIAA* genes under abiotic stress and different tissue was obtained by BLASTn alignment of alfalfa database. Heatmap analysis was performed using the OmicShare tools (http://www.omicshare.com/tools), a free online platform for data analysis. And the expression level is expressed as the calculation results of Z-core normalization for the expression data.

### Protein–protein interaction (PPI) network analysis

As the genomic data of tetraploid cultivated alfalfa were not utilized as pattern data, the homologous genes of *M. truncatula* and *M. sativa* were uploaded to the STRING (v.10.5) database to predict PPI, and the predicted interactions were visualized using Cytoscape software.

### RNA extraction and qRT-PCR analysis

Specific PCR primers of 41 *MsIAA* genes were designed using the NCBI online website Primer-BLAST (http://www.ncbi.nlm.nih.gov/tools/primer-blast) (Table S6). The total RNA was extracted from the 15 samples using an RNA easy Plant Mini Kit according to the manufacturer's instructions (Qiangen, Hilden, Germany). The concentrations of RNA were then detected using an Ultra Micro UV Spectrophotometer (Beijing Dinghaoyuan Biotechnology Co., Ltd., Beijing, China). The total RNA was then reverse-transcribed into cDNA using a Prime Script RT reagent Kit with a gDNA Eraser (Perfect Real Time) (TaKaRa, Japan) and diluted 20 times as template. The qRT-PCR was performed with StepOnePlus™ Real-Time PCR System (ABI) using SYBR premix Ex Taq (Takara, Japan). Triplicate q-PCR of each sample was performed. The reaction volume was 20 μL and included 10 μL of 2 × SuperReal PreMix Plus, 3 μL of ddH_2_O, 5 μL of cDNA template, and 2 μL of forward and reverse primer. The total reaction conditions included a pre-denaturation step at 95℃ for 15 min, 40 cycles of PCR amplification (denaturation at 95℃ for 10 s and annealing at 58℃ for 30 s). The relative expression levels of the selected genes were calculated by 2^−ΔΔCT^ method [73], and normalized to the expression levels of the alfalfa *Actin* gene (AES78237. 1).

### Data analysis

Gene expression level data obtained by qRT-PCR were graphed using the GraphPad Prism 8.0.2. software.

### Supplementary Information

**Supplementary Materials 1.**

## Data Availability

All data generated or analyzed in this study are included in this published article and its supplementary material. The draft genome data of autotetraploid cultivated ("Zhongmu No.1") alfalfa was obtained from fgshare (https://fgshare.com/projects/whole_genome_sequencing_and_assembly_of_Medicago_sativa/66380). The *Arabidopsis* and rice ARF protein sequences were all downloaded from Plant Transcription Factor Database (http://planttfdb.gaolab.org/). Soybean AUX_IAA protein sequence reference Singh et. al. [44], and *M. truncatula* Aux_IAA protein sequence reference Liu *et. al.* [9]. Genome-wide transcriptome data of diferent alfalfa tissues were acquired from the Medicago Analysis Portal (https://medicago.legumeinfo.org/). All transcriptome sequencing data analysed during the current study are available in the NCBI SRA repository (https://www.ncbi.nlm.nih.gov/sra/): SRR7091780-SRR7091794 (cold treatment), SRR7160322-SRR7160357 (ABA, mannitol and salt treatments), SRR22519684-SRR22519695 (aluminum treatment), and SRR5279707-SRR5279711 (lead treatment).
